# Twilight Sleep

**Published:** 1915-12

**Authors:** 


### Twilight Sleep.

Freed from ethical and sentimental complications, the problem of Twilight Sleep is reduced to the following: Can morphine or one of its modifications, plus one or more drugs for "crossed action," produce adequate anaesthesia for a condition fairly comparable with regard to pain and shock, to a fracture, dislocation, forced dilatation of a passage, and even some cutting operations? In the literal, immediate sense, this question can be answered in the affirmative, but the corollary presents itself, whether dosage can be regulated so as to be safe and efficient, considering that the compensating stimulation of pain is likely to be suddenly removed at any time within wide, extremely variable and unforeseeable limits. It is scarcely necessary to state that this problem includes not only the immediate physiologic action of the drug on the vital centers, but its direct and indirect effect on intrinsic vascular and uterine muscle with reference to haemorrhage, its influence on lactation, etc. Further, we must consider whether the drugs which are absorbed and circulate freely and which easily pass through animal membranes, can be so managed as to prevent their toxic action on the child, either by way of the maternal blood or the milk, remembering that morphine is a drug to which infants are peculiarly susceptible, both quantitatively and qualitatively, and that the newly born infant is always liable to respiratory failure to a degree rarely encountered in the adult except under obvious and crude conditions of disease, poisoning and accident.

These are a priori objections, but they rest on established empiric facts and physiologic principles. Can practical ingenuity and skill overcome them to such an extent as to render Twilight Sleep generally available?

